# A short account of metastatic bone disease

**DOI:** 10.1186/1475-2867-11-24

**Published:** 2011-07-27

**Authors:** Liviu Feller, Beverley Kramer, Johan Lemmer

**Affiliations:** 1 Department of Periodontology and Oral Medicine, School of Oral Health Sciences, Faculty of Health Sciences, University of Limpopo, Medunsa Campus, South Africa; 2 School of Anatomical Sciences, Faculty of Health Sciences, University of Witwatersrand, Johannesburg, South Africa

**Keywords:** bone metastasis, PTHrP, jaw

## Abstract

In adults, bone is the preferential target site for metastases from primary cancers of prostate, breast, lungs and thyroid. The tendency of these cancers to metastasize to bone is determined by the anatomical distribution of the blood vessels, by the genetic profile of the cancer cells and by the biological characteristics of the bone microenvironment that favour the growth of metastatic cells of certain cancers.

Metastases to bone may have either an osteolytic or an ostoblastic phenotype. The interaction in the bone microenvironment between biological factors secreted by metastatic cells, and by osteoblasts and osteoclasts, and the osteolytic and osteoblastic factors released from the organic matrix mediate a vicious cycle characterized by metastatic growth and by ongoing progressive bone destruction. This interaction determines the phenotype of the metastatic bone disease.

## Introduction

If primary cancer cells penetrate the walls of blood or lymphatic vessels and gain access to the blood or lymph stream, they may spread to remote sites, where depending upon their interaction with stromal, endothelial and immune cells in their new microenvironment, they may either die, or survive in a dormant state, or undergo clonal expansion to form a metastatic growth [1]. It is probable that those cells that do form a metastatic growth are cells which possess the cytogenetic profile conferring upon them the potential to initiate and sustain growth in a favourable microenvironment [2].

The gene profile that conveys the potential for metastatic growth may be expressed in certain primary cancer cells early in carcinogenesis, or may be acquired only later in the course of primary cancer progression, as a result of evolution of subclones of cancer cells that have undergone multiple episodes of cytogenetic and epigenetic alteration [1-7].

Cells with a cancer stem cell phenotype which disseminate early in the course of the primary cancer, often remain dormant in their new location for a long time, and become active only if or when their new microenvironment favours growth of the metastatic cancer cells. If the primary cancer is large, it can be not only a source of metastasizing cells, but also of biologically active mediators that promote development of favourable prematastatic niches, where colonization by dormant or newly-arrived metastatic cells, will be supported [2,5,6].

Primary cancers of lung, breast, thyroid and prostrate commonly metastasize to bone, almost invariably by haematogenous spread [8-11]. The genetic and epigenetic evolution of metastatic cells of these primary cancers, regardless of whether they leave the primary cancer at an early or at a late stage of its growth, confer tropism for bone upon these metastasizing cells, and fitness to occupy metastatic niches in bone [1].

Cells of osteotropic cancers express the chemokine receptor CXCR4 on their surfaces, and respond to chemoatractant signals generated by its receptor ligand, the stromal-derived factor-1 (SDF-1) that is secreted in the bone microenvironment by osteoblasts, fibroblasts, and by haematopoietic stem/progenitor cells and endothelial stem/progenitor cells in the bone marrow stroma; and is strongly expressed in the bone premetastatic niche [12,13]. The growth of micrometastases is promoted by interaction with gene products in the bone microenvironment (chemokines, cytokines, growth factors) [1].

## Preferred osseous sites for cells metastasizing to bone

Metastases to bone more frequently affect long bones, ribs and vertebrae than other parts of the skeleton, most commonly near the trabecular metaphyses. This pattern of distribution is influenced by the slower rate of blood flow in the marrow-rich metaphyses where the increased sinusoidal blood pressure together with the chemoattraction of the chemokine SDF-1 favours adhesion of the CXCR4+ metastatic cells to the endothelium and their subsequent extravasation [14]. SDF-1 also mobilises bone marrow-derived haematopoietic progenitor cells and endothelial progenitor cells to the metastatic niche [15,16].

Endothelial cells of different candidate metastatic tissues express tissue-specific lectins and integrins that preferentially bind type-specific metastatic cells [17]. For example prostate cancer cell adhesion to bone marrow endothelial cells is mediated by α_v_β_3 _integrins [14,15,18]. Extravasation is mediated by proteolytic enzymes such as matrix metalloproteinase-9 and urokinase plasminogen activator, which promote degradation of and increased permeability of the basement membrane [17].

Once within the bone-marrow stromal microenvironment, the metastatic cells migrate to the endosteal bone surfaces where depending on the cancer cell phenotype they either mediate osteoblastic bone deposition or osteoclastic bone resorption through molecular interactions with resident cells and with biological agents [19,20]. It is likely that metastatic cells expressing α_v_β_3 _integrins bind to bone matrix proteins including collagens, fibronectin, vitronectin and osteopontin, establishing the growth of the metastasis within the bone [12]. There is some controversy as to whether the colonizing cells mediate the angiogenesis necessary for their own proliferation or whether they use the existing bone marrow vasculature [19].

## Classification of bone metastases

In bone, depending upon the phenotype of the cells of the primary cancer, the colonizing metastatic cells induce either osteoclast-mediated bone resorption with the development of osteolytic bone lesions; or osteoblast-mediated bone deposition characterized by increased bone density. In some cases, the development of mixed osteoblastic/osteolytic lesions is induced. In metastatic bone lesions of prostate, carcinoid and other neuroendocrine cancers the osteoblastic phenotype generally predominates whereas metastases of lung, kidney and breast cancers are primarily osteolytic [20,21].

Although osteolytic lesions in metastatic bone disease are brought about by osteoclast mediated bone resorption, therapeutically blocking the osteoclastic activity does not lead to reversal of the osteolysis, indicating that impairment of osteoblast function plays some role in the persistence of osteolytic metastatic bone disease [22]. It is recognized that osteoblastic activity associated with metastatic cells also starts with a phase of abnormal bone resorption, and only later in the course of the metastatic bone disease is there a shift to predominantly osteoblastic activity characterized by formation of abnormal disorganized bone concurrently with a marked reduction in osteoclastic activity [20,23]. Ultimately, the effect of the metastatic cells on normal bone remodelling, and how the balance between bone formation and bone resorption is affected, will define the phenotype of the metastatic bone disease [24,25].

Regardless of their phenotype, metastatic bone lesions cause a variety of complications including pain, pathological fractures, compression of the spinal cord and hypercalcemia, all of which contribute to increased morbidity and mortality [11,21,26,27]. Once bone metastases have developed, conservative cure is no longer possible [20].

## Aspects of bone physiology related to metastatic bone disease

Bone mass is maintained by a balance between osteoblast-mediated bone formation and osteoclast-mediated bone resorption [8]. Osteoblasts originate from bone marrow stromal progenitor cells; they synthesize and secrete extracellular organic matrix, and induce the mineralisation of this matrix. On the other hand osteoclasts originate from monocyte/macrophage lineage of precursor cells in the bone marrow; they resorb bone.

Osteoblast differentiation and maturation is brought about by activation of specific transcription factors (RUNX2, osterix) mediated by bone morphogenic proteins and by Wnt signalling pathways [28]. Several proteins essential for bone formation including bone sialoprotein, osteocalcin, alkaline phosphatase and type 1 collagen, are synthesized by osteoblasts. The functioning of osteoblasts is regulated by parathyroid hormone (PTH); by 1,25 dihydroxyvitamin D [1,25(OH_2_)D]; and by growth factors including transforming growth factor β (TGF β), fibroblast growth factors (FGFs), platelet-derived growth factor (PDGF) and insulin-like growth factors (IGFs) [20,29].

Osteoclasts cause bone resorption and their function is regulated by multiple biological mediators including parathyroid hormone (PTH), calcitonin, oestrogen, 1,25(OH_2_)D, marcrophage colony-stimulating factor (M-CSF), tumour necrosis factor (TNF), interleukin (IL)-1, IL-6, IL-11 and interferon γ [28-30].

The differentiation, maturation and functional activation of osteoclasts is critically dependent on the RANK/RANKL/OPG signalling pathway. Osteoclasts and their precursor cells express the cell surface-receptor RANK. On the other hand osteoblasts precursors and other bone stromal cells express RANK ligand (RANKL), both as a membrane-bound or as a soluble ligand. They also express osteoprotegerin (OPG), a soluble decoy receptor for RANKL [28,31,32].

RANKL binds to RANK on osteoclast progenitor cells stimulating their differentiation, maturation and activation, whereas OPG binds to RANK, inhibiting osteoclastogenenesis [33]. The balance between the activity of RANKL, OPG and the biological mediators listed above determines thus the magnitude of osteoclastogenesis and the subsequent bone resorption [28,34].

Osteoclast differentiation besides being supported by RANKL, is also supported by colony stimulating factor-1 (CSF-1) which is also required for osteoclast survival [35]. Both cell surface-bound and secreted CSF-1 can contribute to osteoclast activation. In an *in vitro *study, high levels of CSF-1 produced by tumour cells protect osteoclasts from the suppressive effects of TGF-β and thus contributed to osteoclast development and survival in bone metastasis [36].

## Osteolytic bone metastasis

Metastases of primary cancers known to cause osteolytic lesions, specifically breast cancer, secrete biological mediators including parathyroid hormone-related protein (PTHrP), interleukin (IL)-11, IL-8, and IL-6 [19,37], which induce osteoclast mediated bone resorption through activation of the RANK/RANKL/OPG signalling pathway [20]. These mediators upregulate the expression of RANKL and down regulate the expression of OPG by osteoblasts and other stromal cells, thus promoting osteoclast differentiation and activation, culminating in bone resorption.

In turn, growth factors, in particular transforming growth factor (TGF)-β and insulin growth factors (IGFs), are released from the organic matrix following the osteoclast mediated dissolution of the inorganic crystalline apatite component of bone, further inducing production of PTHrP which is important for the establishment and progression of osteolytic bone metastasis [38]. This induces upregulation of IL-11 gene expression and promotes tumour cell proliferation and survival. Furthermore, these biological factors provide chemoattractant signals, thus increasing the fitness of the niche to accommodate additional metastatic cells.

Thus, the molecular interaction between cancer cells, stromal cells, and the local 'fertile' microenvironment within the metastatic niche in the bone, creates a 'vicious cycle' that favours osteolytic bone destruction and metastatic growth [2,6,9,19,20,31,37,39].

In addition, certain metastatic cancer cells secrete matrix metalloproteinases (MMP)-2 and MMP-9 [38], as well as osteopontin and bone sialoprotein into the local bone microenvironment. These have the capacity to alter bone turnover and to promote metastatic growth [25,40,41].

Interrupting the RANK/RANKL/OPG signalling pathway by blocking RANKL activity either by OPG or by RANKL specific antibodies; or by blocking RANK receptors with specific antibodies, may bring about inhibition of osteoclast differentiation, maturation and functional activity, resulting in reduced osteoclast-mediated bone resorption with consequent reduction in metastatic growth [30,33].

There is another mechanism associated with the development of osteolytic bone metastases. Primary cancer cells which enter the blood-stream, stimulate peripheral blood mononuclear cells, particularly T lymphocytes, to release tumour necrosis factor-α (TNF-α) which promotes the differentiation of monocytes into osteoclast precursor cells. These osteoclast precursor cells are drawn to the metastatic niche in the bone microenvironment, where they mature and participate in osteoclastic bone resorption, subsequently mediating metastatic growth [10].

## Osteoblastic bone metastasis

The metastaic cells of primary cancers, in particular cells of prostate cancer which have the potential to induce osteoblastic activity, express a number of factors that mediate bone deposition: platelet derive growth factor (PDGF), ligands of the Wnt signalling pathway, insulin-like growth factor (IGF), transforming growth factor-β (TGF-β), bone morphogenic proteins (BMPs), and fibroblast growth factors (FGFs). These promote osteoblast differentiation, proliferation and formation of weak disorganized woven bone [20,21,25,37]. Osteoblastic bone metastasis despite its ultimate manifestation, is preceded or accompanied by osteoclastic activity which plays an important role in preparing the local environment favourable of developing an osteoblastic lesion [21,42].

In the bone microenvironment, BMP-6 promotes osteoblastogenesis and metastatic growth presumably through activation of the downstream target gene Id-1 and activation of MMPs [43]; and BMP-6 is associated with aggressive behaviour of the metastatic bone disease [23,25].

Prostate metastatic cells also express non-collagenous matrix proteins including osteocalcin and bone sialoprotein, which may enhance bone mineralization, and mediate adhesion of osteoclasts and osteoblasts to the hydroxyapatite. The outcome of this is an increase in bone turnover and a net increase in bone formation [44].

Furthermore, prostate cancer cells express the serine proteases kallikrein and urokinase-type plasminogen activator (u-PH) which are mitogenic for osteoblasts; express the prostate-specific antigen (PSA), another serine protease, which has the capacity to stimulate osteoblast activating factors, IGF-1 and TGF-β; and also express the vasoactive peptide endothelin-1 (ET-1) that is a potent vasoconstrictor, but can also induce osteoblast growth [8,20,25,29,31,37].

Prostate cancer cells also produce PTHrP. Although PTHrP is an osteolytic factor, PSA can cleave PTHrP at the N-terminal, not only blocking PTHrP-induced bone resorption, but converting PTHrP to an anabolic stimulant of bone deposition [20,31,37].

The activation of the Wnt canonical pathway in osteoblasts results in bone formation and inhibition of bone resorption [32]. On the other hand, loss of function of Wnt proteins, or upregulation of DKK1, an inhibitor of the Wnt canonical pathway results in a net reduction of bone mass [31,32]. ET-1 which is expressed in bone osteoblastic lesions, decreases DKK-1 expression leading to loss of inhibition of Wnt signalling pathways, resulting in increased bone formation [37]. Furthermore, cells of advanced metastatic prostate carcinoma secrete Wnt ligands, and the Wnt canonical signalling pathway is activated in osteoblasts within the osteoblastic bone lesions [8,24].

## Treatment of metastatic bone disease

Traditionally, the treatment of cancer metastasis comprised chemotherapeutic cytotoxic agents or radiation which directly targets tumour cells. However, as it is evident that the development and progression of metastatic bone disease is driven by complex interactions between metastatic cancer cells and the bone microenvironment, in principle each pathway of these interactions may be an additional potential target for treatment [45].

Bisphosphonates are synthetic analogues of inorganic pyrophosphates which target and inhibit cellular mechanisms of osteoclast-mediated bone resorption, resulting in suppression of bone-turnover and remodeling. Because of these properties, these powerful chemical agents are now routinely used to stabilize bone in the management of metastatic bone disease [20,45,46].

As the RANK/RANKL/OPG signaling pathways are critical for bone turnover, blocking either RANK/RANKL signaling pathways by anti-RANKL neutralizing monoclonal antibodies or by anti-RANK monoclonal antibodies, or by direct application of recombinant OPG-protein, may lead to inhibition of osteoclast differentiation, maturation and functional activity, resulting in suppression of osteoclast-mediated bone resorption [30]. Indeed, a monoclonal antibody blocking agent which binds to RANKL is already in use in treating metastatic bone disease [33,47].

Likewise agents that have the capacity to neutralize or reduce the production of PTHrH, TGF-β or to block the receptors of endothelin-A or TGF-β receptor 1 kinase, can inhibit the development and progression of metastatic bone disease, and are currently under investigation for clinical use [20,37,45].

## Biochemical markers of bone formation

Biochemical markers of bone formation include bone-specific alkaline phosphatase, osteocalcin and C- and N-terminal propeptide of type 1 procollagen; and those of bone resorption include hydroxyproline, pyridinoline, deoxypyridoline, C- and N-terminal crosslinked telopeptide of type 1 collagen, and bone sialoprotein [48-52].

As bone metastases disrupt normal bone remodeling, many biochemical markers of bone turnover are elevated in patients with metastatic bone disease regardless of whether the metastases are predominantly osteolytic or osteoblastic [48-52]. With regard to bone formation markers, the level of bone-specific alkaline phosphatase is usually increased in advanced metastatic bone disease reflecting active repair in an osteolytic lesion, or the presence of an osteoblastic lesion [51]. Of the bone resorption markers, N-telopeptide of type 1 collagen correlates well with the presence and extent of bone metastases and with the prognosis and response to treatment [49].

None of the biochemical markers of bone turnover can monitor the development and progression of metastatic bone disease as accurately as do well established bone imaging techniques [53]. When used alone, they do not possess sufficient diagnostic and prognostic specificity to assess the overall treatment outcome for patients with cancer [50]. However, in combination with other diagnostic techniques and prognostic markers, the biochemical markers of bone turnover are useful tools in the assessment cancer with metastatic bone disease [48-53].

## Metastases to the jaws

Metastases to bone frequently affect the long bones, ribs and the vertebrae, but metastases to the jaws are uncommon. Cancers from almost any primary site can metastasize to the jaws, but those from the breast, lung, kidney and prostate do so most frequently. Jaw metastases occur with equal frequency in males and females; the mandible is more often affected than the maxilla [54-59]. In males, primary cancer of the lung most commonly metastasize to the jaws followed in frequency by prostatic and renal cancers. In females, jaw metastasis most commonly originates from breast primaries followed by primaries of the adrenal and genitalia [56]. Not infrequently a metastasis to the jaw is the first indication of the existence of an undiagnosed primary cancer [54-56].

It is not clear why the jaws are less affected by metastases of osteotropic primary cancers than other bones. However, it may be owing to the fact that the jaws are not rich in hematopoetically active red bone marrow where sinusoidal vascular spaces favour the establishment of metastasis. Moreover breast cancer and prostate cancer often occur in older people in whom the bone marrow of the jaw is even more reduced [54]. It is probable that the mandible is more affected by bone metastatic growth than the maxilla because of the pattern of its blood supply, and because the mandibular retromolar area, the jaw site most affected by metastases, has more red bone marrow than other jaw sites [60].

## Summary

Metastasis of primary cancer cells to bone and subsequent metastatic bone growth is a complex process regulated by multiple signalling pathways and characterized by molecular interactions between the metastatic cells and the extracellular and cellular components of the bone microenvironment. These interactions determine whether the metastatic bone disease will be predominantly osteolytic or osteoblastic.

## Competing interests

The authors declare that they have no competing interests.

## Authors' contributions

The concept of the paper was devised by LF. LF, BK and JL contributed equally to the intellectual input of the paper. All authors read and approved the final manuscript.

## References

[B1] Kang Y Siegel PM Shu W Drobnjak M Kakonen SM Cordon-Cardo C Guise TA Massague J A multigenic program mediating breast cancer metastasis to bone Cancer Cell 2003 3 537 549 10.1016/S1535-6108(03)00132-6 12842083

[B2] Chiang AC Massague J Molecular basis of metastasis N Engl J Med 2008 359 2814 2823 10.1056/NEJMra0805239 19109576 PMC4189180

[B3] Minn AJ Kang Y Serganova I Gupta GP Giri DD Doubrovin M Ponomarev V Gerald WL Blasberg R Massague J Distinct organ-specific metastatic potential of individual breast cancer cells and primary tumors J Clin Invest 2005 115 44 55 15630443 10.1172/JCI22320 PMC539194

[B4] Schmidt-Kittler O Ragg T Daskalakis A Granzow M Ahr A Blankenstein TJ Kaufmann M Diebold J Arnholdt H Muller P Bischoff J Harich D Schlimok G Riethmuller G Eils R Klein CA From latent disseminated cells to overt metastasis: genetic analysis of systemic breast cancer progression Proc Natl Acad Sci USA 2003 100 7737 7742 10.1073/pnas.1331931100 12808139 PMC164657

[B5] Husemann Y Geigl JB Schubert F Musiani P Meyer M Burghart E Forni G Eils R Fehm T Riethmüller G Klein CA Systemic spread is an early step in breast cancer Cancer Cell 2008 13 58 68 10.1016/j.ccr.2007.12.003 18167340

[B6] Nguyen DX Bos PD Massague J Metastasis: from dissemination to organ-specific colonization Nat Rev Cancer 2009 9 274 284 10.1038/nrc2622 19308067

[B7] Feller L Bouckaert M Chikte UM Wood NH Khammissa RA Meyerov R Lemmer J A short account of cancer--specifically in relation to squamous cell carcinoma SADJ 2010 65 322 324 21133236

[B8] Logothetis CJ Lin SH Osteoblasts in prostate cancer metastasis to bone Nat Rev Cancer 2005 5 21 28 10.1038/nrc1528 15630412

[B9] Nguyen DX Massague J Genetic determinants of cancer metastasis Nat Rev Genet 2007 8 341 352 17440531 10.1038/nrg2101

[B10] Roato I Grano M Brunetti G Colucci S Mussa A Bertetto O Ferracini R Mechanisms of spontaneous osteoclastogenesis in cancer with bone involvement FASEB J 2005 19 228 230 15550550 10.1096/fj.04-1823fje

[B11] Major PP Cook RJ Lipton A Smith MR Terpos E Coleman RE Natural history of malignant bone disease in breast cancer and the use of cumulative mean functions to measure skeletal morbidity BMC Cancer 2009 9 272 10.1186/1471-2407-9-272 19660124 PMC2739221

[B12] Sun YX Fang M Wang J Cooper CR Pienta KJ Taichman RS Expression and activation of alpha v beta 3 integrins by SDF-1/CXC12 increases the aggressiveness of prostate cancer cells Prostate 2007 67 61 73 10.1002/pros.20500 17034033

[B13] Wels J Kaplan RN Rafii S Lyden D Migratory neighbors and distant invaders: tumor-associated niche cells Genes Dev 2008 22 559 574 10.1101/gad.1636908 18316475 PMC2731657

[B14] Wang J Loberg R Taichman RS The pivotal role of CXCL12 (SDF-1)/CXCR4 axis in bone metastasis Cancer Metastasis Rev 2006 25 573 587 17165132 10.1007/s10555-006-9019-x

[B15] Taichman RS Loberg RD Mehra R Pienta KJ The evolving biology and treatment of prostate cancer J Clin Invest 2007 117 2351 2361 10.1172/JCI31791 17786228 PMC1952634

[B16] Kaplan RN Rafii S Lyden D Preparing the "soil": the premetastatic niche Cancer Res 2006 66 11089 11093 10.1158/0008-5472.CAN-06-2407 17145848 PMC2952469

[B17] Pienta KJ Loberg R The "emigration, migration, and immigration"of prostate cancer Clin Prostate Cancer 2005 4 24 30 15992458 10.3816/cgc.2005.n.008

[B18] Sun YX Schneider A Jung Y Wang J Dai J Cook K Osman NI Koh-Paige AJ Shim H Pienta KJ Keller ET McCauley LK Taichman RS Skeletal localization and neutralization of the SDF-1(CXCL12)/CXCR4 axis blocks prostate cancer metastasis and growth in osseous sites in vivo J Bone Miner Res 2005 20 318 329 15647826 10.1359/JBMR.041109

[B19] Guise TA Kozlow WM Heras-Herzig A Padalecki SS Yin JJ Chirgwin JM Molecular mechanisms of breast cancer metastases to bone Clin Breast Cancer 2005 5 S46 S53 10.3816/CBC.2005.s.004 15807924

[B20] Mundy GR Metastasis to bone: causes, consequences and therapeutic opportunities Nat Rev Cancer 2002 2 584 593 10.1038/nrc867 12154351

[B21] Schwartz GG Prostate cancer, serum parathyroid hormone, and the progression of skeletal metastases Cancer Epidemiol Biomarkers Prev 2008 17 478 483 10.1158/1055-9965.EPI-07-2747 18349265

[B22] Tian E Zhan F Walker R Rasmussen E Ma Y Barlogie B Shaughnessy JD Jr The role of the Wnt-signaling antagonist DKK1 in the development of osteolytic lesions in multiple myeloma N Engl J Med 2003 349 2483 2494 10.1056/NEJMoa030847 14695408

[B23] Dai J Keller J Zhang J Lu Y Yao Z Keller ET Bone morphogenetic protein-6 promotes osteoblastic prostate cancer bone metastases through a dual mechanism Cancer Res 2005 65 8274 8285 10.1158/0008-5472.CAN-05-1891 16166304

[B24] Li ZG Yang J Vazquez ES Rose D Vakar-Lopez F Mathew P Lopez A Logothetis CJ Lin SH Navone NM Low-density lipoprotein receptor-related protein 5 (LRP5) mediates the prostate cancer-induced formation of new bone Oncogene 2008 27 596 603 10.1038/sj.onc.1210694 17700537

[B25] Keller ET Zhang J Cooper CR Smith PC McCauley LK Pienta KJ Taichman RS Prostate carcinoma skeletal metastases: cross-talk between tumor and bone Cancer Metastasis Rev 2001 20 333 349 10.1023/A:1015599831232 12085970

[B26] Brown JE Coleman RE The present and future role of bisphosphonates in the management of patients with breast cancer Breast Cancer Res 2002 4 24 29 11879556 10.1186/bcr413 PMC138712

[B27] Brown JE Neville-Webbe H Coleman RE The role of bisphosphonates in breast and prostate cancers Endocr Relat Cancer 2004 11 207 224 10.1677/erc.0.0110207 15163299

[B28] Bringhurst F Demay M Krane S Kronenberg H Kasper D, Hauser S, Longo D, Jameson J, Loscaizo J Bone and Mineral Metabolism in Health and Disease Harrison's Principles of Internal Medicine 2008 17 New York: McGraw-Hill 2365 237621796030

[B29] Koeneman KS Yeung F Chung LW Osteomimetic properties of prostate cancer cells: a hypothesis supporting the predilection of prostate cancer metastasis and growth in the bone environment Prostate 1999 39 246 261 10.1002/(SICI)1097-0045(19990601)39:4<246::AID-PROS5>3.0.CO;2-U 10344214

[B30] Bai YD Yang FS Xuan K Bai YX Wu BL Inhibition of RANK/RANKL signal transduction pathway: a promising approach for osteoporosis treatment Med Hypotheses 2008 71 256 258 10.1016/j.mehy.2008.03.021 18445511

[B31] Roodman GD Mechanisms of bone metastasis N Engl J Med 2004 350 1655 1664 10.1056/NEJMra030831 15084698

[B32] Glass DA Bialek P Ahn JD Starbuck M Patel MS Clevers H Taketo MM Long F McMahon AP Lang RA Karsenty G Canonical Wnt signaling in differentiated osteoblasts controls osteoclast differentiation Dev Cell 2005 8 751 764 10.1016/j.devcel.2005.02.017 15866165

[B33] Romas E Clinical applications of RANK-ligand inhibition Intern Med J 2009 39 110 116 10.1111/j.1445-5994.2008.01732.x 19356186

[B34] Fenton R Longo D Kasper D, Hauser S, Longo D, Jameson J, Loscaizo J Cancer Cell Biology and Angiogenesis Harrison's Principles of Internal Medicine 2008 17 New York: McGraw-Hill 498 51321796030

[B35] Biskobing DM Fan X Rubin J Characterization of MCSF-induced proliferation and subsequent osteoclast formation in murine marrow culture J Bone Miner Res 1995 10 1025 1032 7484277 10.1002/jbmr.5650100706

[B36] Yagiz K Rittling SR Both cell-surface and secreted CSF-1 expressed by tumor cells metastatic to bone can contribute to osteoclast activation Exp Cell Res 2009 315 2442 2452 10.1016/j.yexcr.2009.05.002 19427849 PMC2704245

[B37] Guise TA Mohammad KS Clines G Stebbins EG Wong DH Higgins LS Vessella R Corey E Padalecki S Suva L Chirgwin JM Basic mechanisms responsible forb osteolytic and osteoblastic bone metastases Clin Cancer Res 2006 12 6213s 6216s 10.1158/1078-0432.CCR-06-1007 17062703

[B38] Guise TA Molecular mechanisms of osteolytic bone metastases Cancer 2000 88 2892 2898 10.1002/1097-0142(20000615)88:12+<2892::AID-CNCR2>3.0.CO;2-Y 10898330

[B39] Yoneda T Cellular and molecular basis of preferential metastasis of breast cancer to bone J Orthop Sci 2000 5 75 81 10.1007/s007760050012 10664443

[B40] Lelekakis M Moseley JM Martin TJ Hards D Williams E Ho P Lowen D Javni J Miller FR Slavin J Anderson RL A novel orthotopic model of breast cancer metastasis to bone Clin Exp Metastasis 1999 17 163 170 10.1023/A:1006689719505 10411109

[B41] Jacob K Webber M Benayahu D Kleinman HK Osteonectin promotes prostate cancer cell migration and invasion: a possible mechanism for metastasis to bone Cancer Res 1999 59 4453 4457 10485497

[B42] Roato I D'Amelio P Gorassini E Grimaldi A Bonello L Fiori C Delsedime L Tizzani A De Libero A Isaia G Ferracini R Osteoclasts are active in bone forming metastases of prostate cancer patients PloS one 2008 3 e3627 10.1371/journal.pone.0003627 18978943 PMC2574033

[B43] Darby S Cross SS Brown NJ Hamdy FC Robson CN BMP-6 over-expression in prostate cancer is associated with increased Id-1 protein and a more invasive phenotype J Pathol 2008 214 394 404 10.1002/path.2292 18072288

[B44] Chung LW Baseman A Assikis V Zhau HE Molecular insights into prostate cancer progression: the missing link of tumor microenvironment J Urol 2005 173 10 20 10.1097/01.ju.0000141582.15218.10 15592017

[B45] Loberg RD Logothetis CJ Keller ET Pienta KJ Pathogenesis and treatment of prostate cancer bone metastases: targeting the lethal phenotype J Clin Oncol 2005 23 8232 8241 10.1200/JCO.2005.03.0841 16278478

[B46] Feller L Wood NH Khammissa RA Chikte UM Bouckaert M Lemmer J Bisphosphonate-related osteonecrosis of the jaw SADJ 2011 66 30 32 21510174

[B47] Barton MK Denosumab an option for patients with bone metastasis from breast cancer CA Cancer J Clin 2011 61 135 136 10.3322/caac.20116 21532096

[B48] Coleman R Brown J Terpos E Lipton A Smith MR Cook R Major P Bone markers and their prognostic value in metastatic bone disease: clinical evidence and future directions Cancer Treat Rev 2008 34 629 639 10.1016/j.ctrv.2008.05.001 18579314 PMC3090688

[B49] Brown JE Cook RJ Major P Lipton A Saad F Smith M Lee KA Zheng M Hei YJ Coleman RE Bone turnover markers as predictors of skeletal complications in prostate cancer, lung cancer, and other solid tumors J Natl Cancer Inst 2005 97 59 69 10.1093/jnci/dji002 15632381

[B50] Seibel MJ Clinical use of markers of bone turnover in metastatic bone disease Nat Clin Pract Oncol 2005 2 504 517 16205770 10.1038/ncponc0320

[B51] Seibel MJ The use of molecular markers of bone turnover in the management of patients with metastatic bone disease Clin Endocrinol 2008 68 839 849 10.1111/j.1365-2265.2007.03112.x 17980010

[B52] Cremers S Garnero P Biochemical markers of bone turnover in the clinical development of drugs for osteoporosis and metastatic bone disease: potential uses and pitfalls Drugs 2006 66 2031 2058 10.2165/00003495-200666160-00001 17112299

[B53] Huang Q Ouyang X Biochemical-markers for the diagnosis of bone metastasis: A review in clinical Cancer Epidemiol 2011 10.1016/j.canep.2011.02.001 21474411

[B54] Hirshberg A Leibovich P Buchner A Metastatic tumors to the jawbones: analysis of 390 cases J Oral Pathol Med 1994 23 337 341 10.1111/j.1600-0714.1994.tb00072.x 7815371

[B55] D'Silva NJ Summerlin DJ Cordell KG Abdelsayed RA Tomich CE Hanks CT Fear D Meyrowitz S Metastatic tumors in the jaws: a retrospective study of 114 cases J Am Dent Assoc 2006 137 1667 1672 17138711 10.14219/jada.archive.2006.0112

[B56] Hirshberg A Shnaiderman-Shapiro A Kaplan I Berger R Metastatic tumours to the oral cavity - pathogenesis and analysis of 673 cases Oral Oncol 2008 44 743 752 10.1016/j.oraloncology.2007.09.012 18061527

[B57] Irani S Metastasis to head and neck area: a 16-year retrospective study Am J Otolaryngol 2011 32 24 27 10.1016/j.amjoto.2009.09.006 20031269

[B58] van der Waal RI Buter J van der Waal I Oral metastases: report of 24 cases Br J Oral Maxillofac Surg 2003 41 3 6 10.1016/S0266-4356(02)00301-7 12576032

[B59] Ertas U Yalcin E Erdogan F Invasive ductal carcinoma with multiple metastases to facial and cranial bones: a case report Eur J Dent 2010 4 334 337 20613924 PMC2897869

[B60] Katsnelson A Tartakovsky JV Miloro M Review of the literature for mandibular metastasis illustrated by a case of lung metastasis to the temporomandibular joint in an HIV-positive patient J Oral Maxillofac Surg 2010 68 1960 1964 10.1016/j.joms.2009.07.075 20381938

